# Application of Cycloaddition Reactions to the Syntheses of Novel Boron Compounds

**DOI:** 10.3390/molecules15129437

**Published:** 2010-12-21

**Authors:** Yinghuai Zhu, Xiao Siwei, John A. Maguire, Narayan S. Hosmane

**Affiliations:** 1 Institute of Chemical and Engineering Sciences, No. 1 Pesek Road, Jurong Island 627833, Singapore; 2 Department of Chemistry, Southern Methodist University, Dallas, Texas 75275, USA; 3 Department of Chemistry and Chemical Biology, Northern Illinois University, DeKalb, Illinois 60115, USA

**Keywords:** cycloaddition reaction, carborane, boron neutron capture therapy, boron-based material, boron compounds, cross-coupling reaction, boron reagent

## Abstract

This review covers the application of cycloaddition reactions in forming the boron-containing compounds such as symmetric star-shaped boron-enriched dendritic molecules, nano-structured boron materials and aromatic boronic esters. The resulting boron compounds are potentially important reagents for both materials science and medical applications such as in boron neutron capture therapy (BNCT) in cancer treatment and as drug delivery agents and synthetic intermediates for carbon-carbon cross-coupling reactions. In addition, the use of boron cage compounds in a number of cycloaddition reactions to synthesize unique aromatic species will be reviewed briefly.

## 1. Introduction

It is well recognized that cycloaddition reactions are among the most powerful and commonly used methodologies in organic synthesis to construct various molecules which are important in industry and academia. The definition, classification and extremely extensive application of cycloadditions have been well documented [1,2]. Numerous successful examples such as [2 + 2], [2 + 3] and [2 + 4] cycloaddition reactions appear in the literature [1,2]. Unlike the ionic or free radical reactions, cycloaddition reactions are relatively unaffected by solvent changes, the presence of radical initiators or scavenging reagents, or by nucleophilic or electrophilic catalysts [1,2]. Therefore, the typical cycloaddition reaction may be widely applied in forming boron containing small molecules and macromolecules. Since boron compounds possess unique physical and chemical properties, such as high thermal stability and low toxicity, they are among the most important reagents and materials for a wide range of applications in organic transformations as both catalysts and reagents [3,4,5,6,7,8,9,10,11]. Other potentially important applications of boron compounds can be found in medicine, polymer science, recovery of radioactive metals, supramolecular chemistry, and the synthesis of new materials [3,4,5,6,7,8,9,10,11]. To date, the pace of development in boron chemistry is increasing dramatically as new ways are found to construct and apply these remarkable compounds. Although various methodologies have been used to synthesize boron composites [3,4,5,6,7,8,9,10,11], this review will mainly concentrate on the recent developments of preparing boron compounds or materials using cycloaddition reactions. 

In cycloaddition, “two or more unsaturated molecules (or parts of the same molecule) combine with the formation of a cyclic adduct in which there is a net reduction of the bond multiplicity” [12]. Based on the concept, cycloadditions are notated as [m + n + …], in which m and n refer to the electrons involved in the cycloaddition (IUPAC) [12]. In this review, three types of the typical cycloaddition patterns: the [2 + 2 + 2] and [2 + 2] cycloadditions; [2 + 3] cycloadditions; and [2 + 4] cycloadditions will be discussed. All the selected examples contain boron species, which are either participant atoms or functional groups. Therefore, the review summarizes the current progresses in synthetic strategy of boron compounds using such cycloaddition reactions. Some applications including drug delivery and coupling reactions of the derived products are also described. 

## 2. [2+2+2] and the [2+2] Cycloadditions

**Scheme 1:**
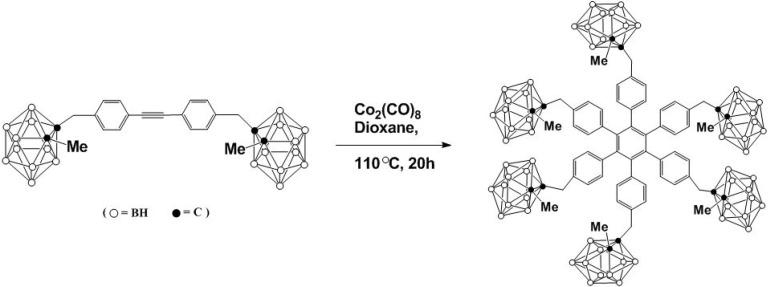
Synthesis of carborane appended star-shaped molecule.

In general, [2 + 2 + 2] cycloaddition reactions are used to construct six-membered rings such as benzene, with transition metal complexes as catalysts [13,14,15,16,17]. Recently, the synthesis of a star-shaped molecule containing multiple carborane clusters involving this type of cycloaddition reaction has been reported [18,19,20]. In the presence of a cobalt catalyst, carborane-functionalized alkynes undergo [2 + 2 + 2] cycloaddition as shown in Scheme 1 to form a dendritic molecule in 62% yield [18]. The boron enriched compound is highly thermally stable with an endothermic peak at 468 °C. Water solubility could be achieved by a decapitation of the attached carborane cages. These water soluble species should prove to be useful precursors for BNCT applications and in catalysis [18]. 

Aromatic boronic esters are the most valuable and heavily used synthetic intermediates in modern organic chemistry due to their ability to participate in functional group transformations and carbon—carbon bond forming reactions [21,22]. In general, these compounds are prepared *via* a functional group interconversion strategy from a starting aryl halide or triflate [23,24,25]. However, the requirement of these precursors can prove problematic when more highly substituted or heavily functionalized boronic ester products are needed.

**Scheme 2:**
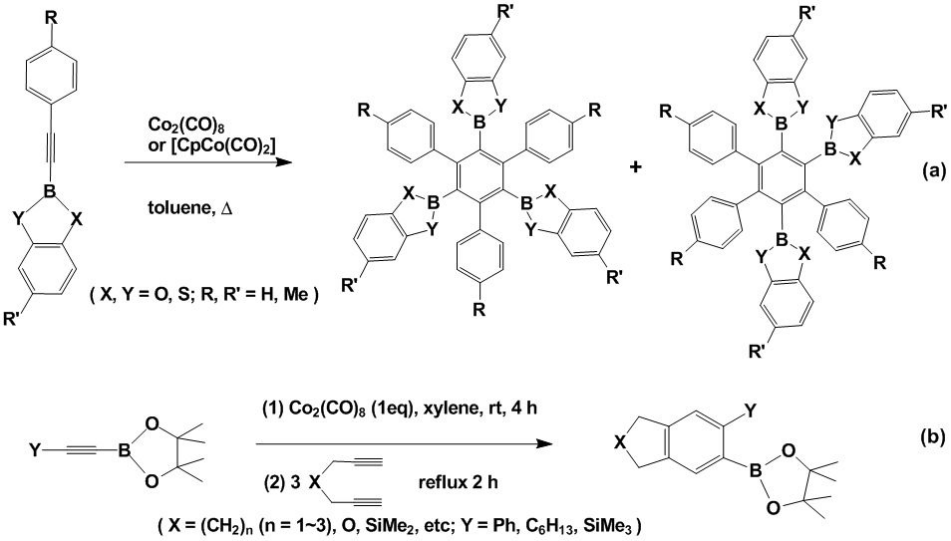
Synthesis of fused arylboronic esters.

Accordingly, much effort has been concentrated on developing novel and efficient methods to synthesize arylboronates and heterocyclic variants, with cycloaddition reactions prevalent among them. Scheme 2 depicts [2 + 2 + 2] cycloaddition reactions of borate functionalized alkynes that lead to the formation of arylboronic esters in reasonable yields [26,27]. With Co_2_(CO)_8_ or CpCo(CO)_2_ as catalyst, alkylcatechol-, thiocatechol- and dithiocatechol-bound boronic esters undergo cyclotrimerization reactions generating the corresponding borylbenzene derivatives. Interestingly, when CpCo(CO)_2_ or CpCo(C_2_H_4_)_2_ was used as catalyst and the reactions were conducted at room temperature, the products resulting from [2 + 2] cycloaddition were isolated in good yields [26]. In addition, [2 + 2] cycloaddition is the most powerful and synthetically useful strategy for construction of four member carbo- and heterocyclic ring systems [2]. All of the resulting arylboronates can undergo carbon-carbon cross-coupling reactions to construct new molecules.

**Scheme 3:**
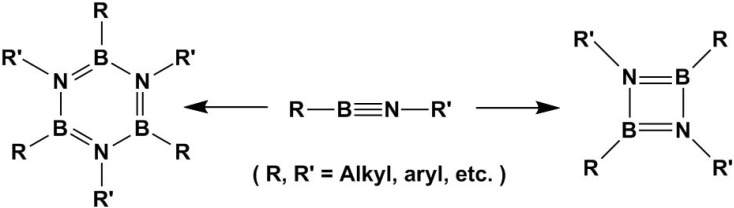
B≡N bond cycloaddition.

Similar to the C≡C bond, the [2 + 2 + 2] and [2 + 2] cycloaddition reactions also have been found to occur between B≡N bonds to form four and six-membered rings, respectively, as shown in Scheme 3 [28,29]. The resulting six-membered borazines, known as inorganic analogues of benzene, have been known for many years. However, very limited work has been done on their applications, except for their use as ceramic boron nitride (BN) precursors [30]. Recently, borazines have attracted renewed interest in material chemistry. As a new class of multifunctional and thermally stable materials with high charge mobilities, borazines may find wide applications in electroluminescent devices [31,32]. The cycloaddition reactions of B≡N are forecasted to play key roles in material chemistry for the synthesis of borazine variants. 

## 3. [2+3] Cycloadditions

**Scheme 4:**
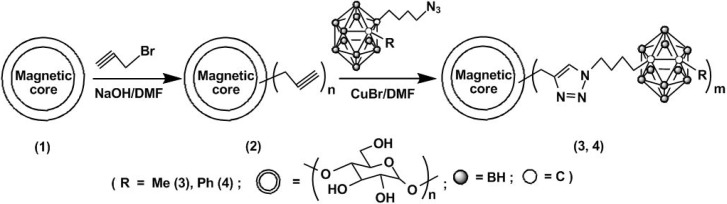
Synthesis of encapsulated magnetic nanocomposites.

The 1,3-dipolar [2 + 3] cycloaddition is a reaction between a 1,3-dipole and a substituted alkene to form a five-membered ring [33]. Catalytic azide-alkyne cycloadditions, known as “click” reactions, are a well recognized [2 + 3] cycloaddition. These [2 + 3] cycloaddition reactions have been widely used in chemical transformations in biological application and materials chemistry such as cell surface labeling, biopolymer-virus conjugation, and block copolymer synthesis [34,35,36,37]. *Ortho*-carborane cages have been successfully attached to modified magnetic nanoparticles *via* catalytic “click” cycloadditions between 1-R-2-butyl-*ortho*-C_2_B_10_H_10_ (R = Me, Ph) and propargyl group-enriched magnetic nanoparticles (Scheme 4). 

A loading amount of 9.83 mmol boron atom/g starch-matrixed magnetic nanoparticles has been achieved for the R = Me compound. The resulting nanocomposites have been found to accumulate in tumor cells in high boron concentrations of 51.4 μg/g tumor and ratios of around 10:1 tumor to normal tissues in the presence of an external magnetic field (1.14T). These results have provided new avenues of research in neutron capture therapy (NCT), with combination of the drugs with BNCT/MRI/Thermotherapy characteristics [38]. 

**Scheme 5:**
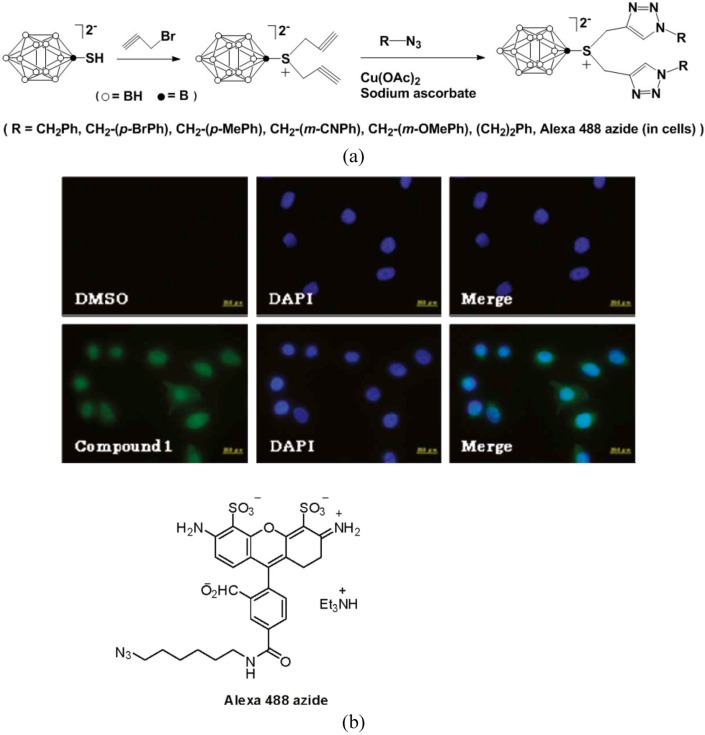
Click chemistry of *S,S*-dipropargyl-SB_12_H_11_^-^, (a) in acetonitrile, (b) in cells.

Nakamura, *et al*., recently utilized the click reaction to functionalize [B_12_H_11_SH]^2-^ (BSH) with organic molecules, both in solvent media and in cells (Scheme 5) [39]. In their study, *S,S*-dipropargyl-SB_12_H_11_^-^ and *S*-propargyl-SB_12_H_11_^2-^ were prepared by reaction of propargyl bromide with [(CH_3_)_4_N]_2_B_12_H_11_SH and [(CH_3_)_4_N]_2_B_12_H_11_S(CH_2_)_2_CN, respectively.

**Scheme 6:**
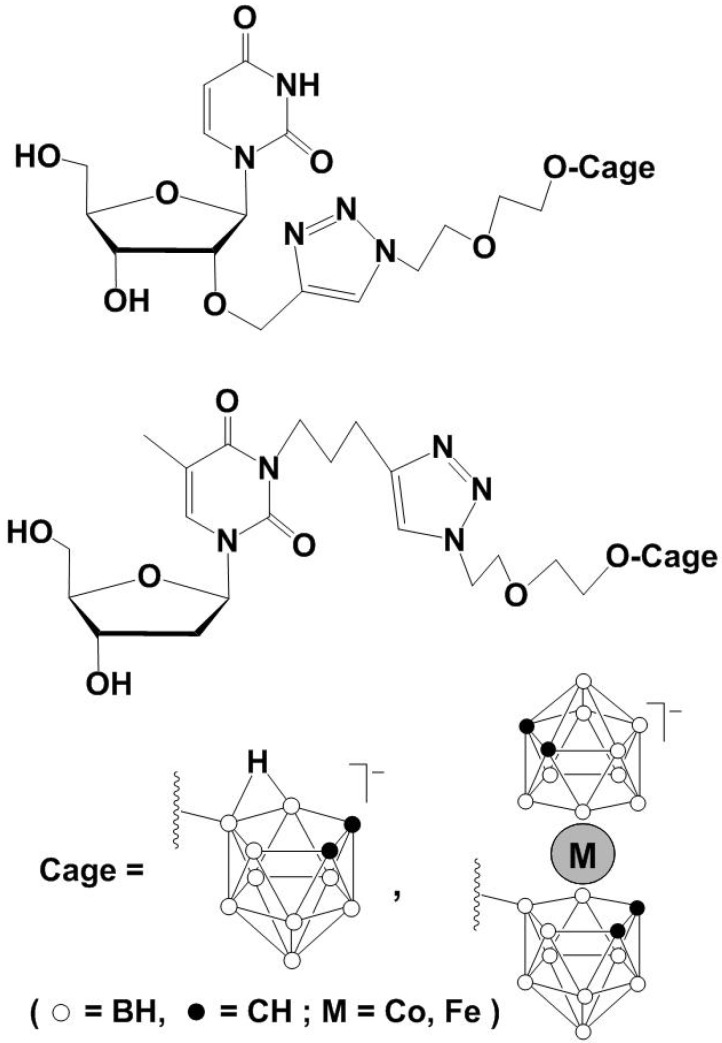
Synthesis of nucleoside-boron cluster conjugates.

These compounds further reacted with various azides catalyzed by Cu(II) ascorbate, to give the corresponding monoanionic bis-triazolo BSH derivatives and dianionic monotriazole BSH derivatives in excellent yields (Scheme 5-a). The click reaction is very useful, not only for the synthesis of various BSH-containing organic compounds for boron neutron capture therapy (BNCT), but also for the visualization of boron clusters in cells. The click cycloaddition reaction of *S,S*-dipropargyl-SB_12_H_11_^-^ with Alexa Fluor 488 azide dye indicated that the product accumulation was not in the cytoplasm, but in the nuclei of HeLa cells as shown in Scheme 5(b) [39]. Using the click methodology, borane clusters and metallacarborane complexes were successfully attached to nucleosides (Scheme 6) [40]. These boron enriched conjugates may find new application in BNCT.

**Scheme 7:**
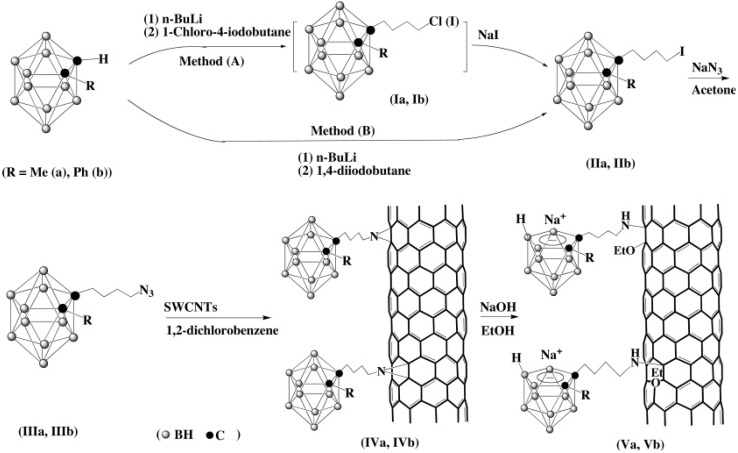
Syntheses of substituted carborane-appended SWCNTs.

Another [2 + 3] cycloaddition reaction occurs between R-N_3_ and C=C bonds to form a five-membered ring. At high temperature, the resulting heteroatom ring decomposes to generate a three-membered ring and release one N_2_ molecule [41]. In organic chemistry, this type of reaction is also called a nitrene cycloaddition reaction [41]. In our lab, substituted *ortho*-carborane cages have been successfully attached to the side walls of single wall carbon nanotubes (SWCNTs) *via* nitrene cycloadditions as shown in Scheme 7 [42]. All of the five-membered ring intermediates, generated from a [2 + 3] cycloaddition between azides and C=C bonds in SWCNTs, are decomposed in our experiments by the long-term refluxing at high temperature. This was confirmed by the absence of a –N=N-N– absorption in their IR spectra and the presence of N_2_ as a product. The decapitations of these C_2_B_10_ carborane cages, with the appended SWCNTs intact, were accomplished by the reaction with sodium hydroxide in refluxing ethanol. During base reflux, the three-membered ring formed by the nitrene and SWCNT was opened to produce water-soluble SWCNTs in which the side walls are functionalized by both substituted *nido*-C_2_B_9_ carborane units and ethoxide moieties. Selected tissue distribution studies on one of these nanotubes, {([Na^+^][1-Me-2-((CH2)4NH-)-1,2-C2B9H10][OEt])_n_(SWCNT)}, show that the boron atoms are concentrated more in tumors cells than in blood and other organs, making them an attractive nanovehicle for the delivery of boron to tumor cells for an effective boron neutron capture therapy in the treatment of cancer [42].

**Scheme 8:**
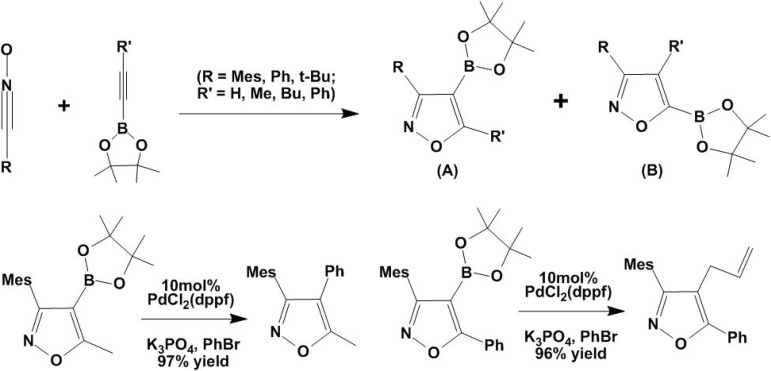
Synthesis of isoxazoleboronic esters.

The [2 + 3] cycloaddition reactions between 1,3-dipolar nitrile oxides and alkynylboronates result in the formation of isoxazoleboronic esters with excellent regioselectivity (Scheme 8) [43]. Specifically, longer chain alkyl and phenyl substituents provided the 4-substituted boronic esters as single regioisomers (A) in good yields (see Scheme 8). These potentially valuable intermediates of organic synthesis have demonstrated good activity and high regioselectivity to conduct Suzuki coupling reactions (see Scheme 8) [43].

## 4. [2+4] Cycloaddition

**Scheme 9:**
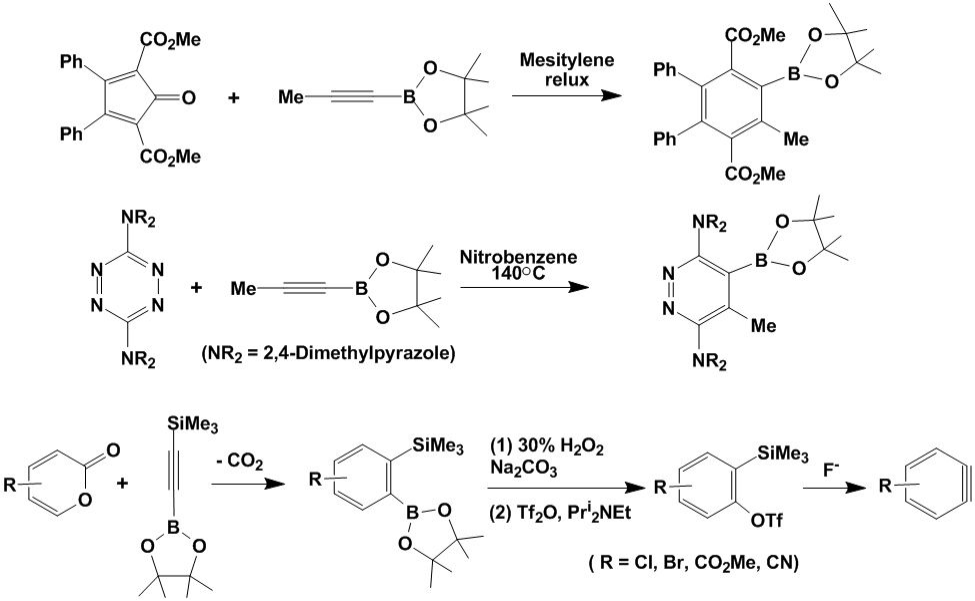
Cycloaddition of alkynylboronates.

The [2 + 4] cycloaddition, known as the Diels-Alder reaction, is the most common cycloaddition reaction to form six-membered rings [1,2]. It requires very little energy without catalyst, and thus has been widely used in many organic reactions [44,45,46]. Alkynylboronates have found to be reactive reagents to undergo the [2 + 4] cycloaddition to form six-membered functionalized organoboronates [47,48]. Harrity, *et al*., found that alkynylboronates are relatively electron rich and thus have a cycloaddition reactivity that resembles that of acetylene. Therefore, the [2 + 4] reactions between alkynylboronates and electron-rich dienes were unsuccessful [47,48]. The use of cyclopentadienones, tetrazines and 2-pyrones as diene components led to the formation of highly functionalized organoboron synthetic intermediates as outlined in Scheme 9. For the trifluoromethylsulfonyl-substituted alkynylboronates, the resulting functionalized benzene can be converted to benzynes that could undergo further organic transformations. 

**Scheme 10:**
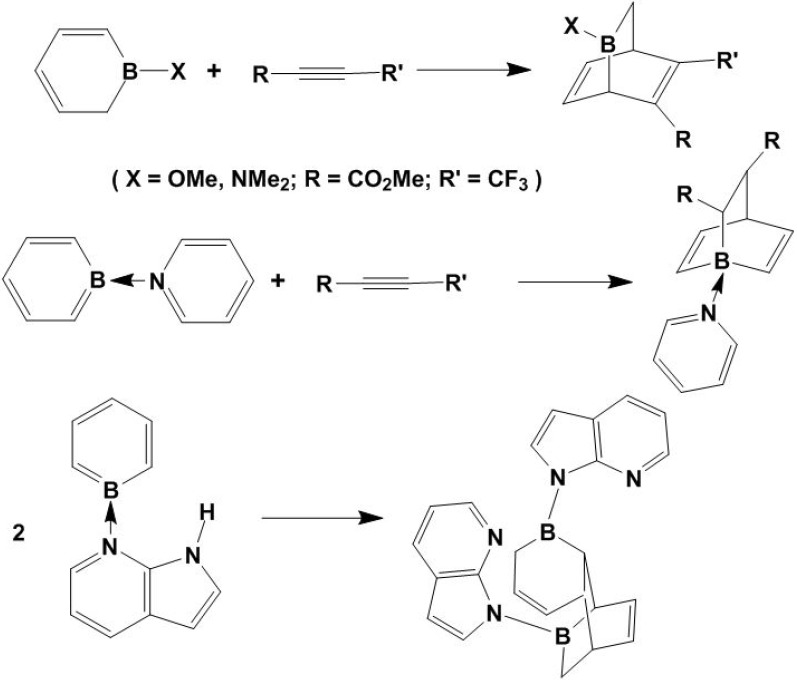
Alkyne-borabenzene and boracyclohexadiene [2+4] cycloaddition.

In the presence of alkyne, boracyclohexadiene also undergoes [2 + 4] cycloaddition to form 7-borabarrelenes, as shown in Scheme 10; no self-dimerization of boracyclohexadiene has been found [49,50,51]. However, the 7-azaindolyl-substituted boracyclohexadiene is unstable and spontaneously undergoes a [2 + 4] cycloaddition to produce a diboratricyclobarrelene cage as shown in Scheme 10 [51]. In the resulting polycyclic variants, the N→B linkage is stable. These compounds containing π-conjugated boron heterocycles may exhibit potentially promising exploitable electro-optical properties [52]. The cyclic azaindole-borabenzenes can also be used as ligands to chelate metals [51]. 

**Scheme 11:**
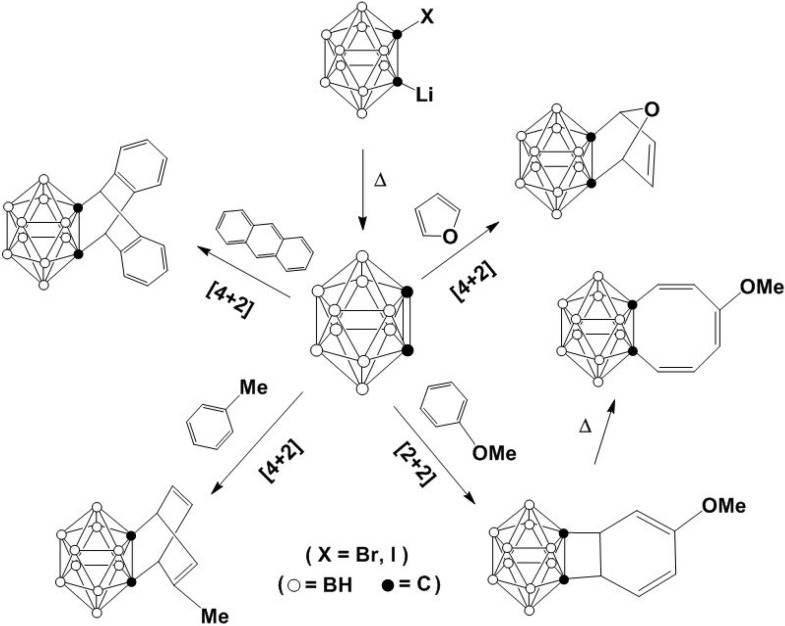
Reactions of carboryne.

Interestingly, boron clusters also have been found to be involved in cycloaddition reactions. The *closo*-carborane is one of the extremely thermally stable clusters, which has been widely investigated for both academic research and potential medical applications [53]. The deprotonation of *closo*-carborane with two equivalent of *n*-BuLi led to the formation of the lithium dianion which reacted further with bromine or iodine to produce the corresponding bromo- or iodo- monoanions [53]. Heating the reaction mixture provided carboryne, which is isolobal with benzyne, as shown in Scheme 11 [54,55,56,57,58]. The carboryne undergoes either [2 + 4] or [2 + 2] reactions with suitable dienes [57,58]. For anisole, it has been confirmed that cyclooctatetraenocarboranes are generated from the thermal rearrangement of [2 + 2] cycloaddition intermediate [58]. Like their pristine precursor, carborane, the derived cyclic compounds can potentially be used as a source of BNCT [59]. They may also be used in structural studies in crystallography.

## 5. Concluding Remarks

Much like carbon, a neighbor element of boron in the Periodic Table, complicated boron-based molecules such as clusters, rings and chains have been constructed. Indeed, the future of boron chemistry is dependent upon the continued fusion of their remarkable chemistry with that of organic synthesis. The review summarizes the latest developments in boron-functionalized building blocks in cycloaddition reactions. As described above, cycloaddition reactions are some of the most important and efficient tools to generate novel boron containing molecules and materials, and thus have great synthetic potential in boron chemistry. The resulting boron composites open wide areas of potential applications, which include: (1) as therapeutic and diagnostic agents in biomedicine, (2) materials such as boron nitride, (3) synthetic intermediates such as aromatic boronic esters, (4) ultrathin films and semi-conductors in nanotechnology, as well as (5) fuel cells in a hydrogen economy. In addition, boron clusters participate in cycloaddition reactions that may form some unique aromatic species which cannot be synthesized by other existing methods. Considering the new introduced C=C bonds in these compounds, it’s reasonable to expect they will undergo further cycloaddition reactions.
